# COVID-19: What we talk about when we talk about masks

**DOI:** 10.1590/0037-8682-0527-2020

**Published:** 2020-11-06

**Authors:** Cristiane Ravagnani Fortaleza, Lenice do Rosário de Souza, Juliana Machado Rúgolo, Carlos Magno Castelo Branco Fortaleza

**Affiliations:** 1 Universidade Estadual Paulista, Faculdade de Medicina de Botucatu, Hospital das Clínicas, Botucatu, SP, Brasil.; 2 Universidade Estadual Paulista, Faculdade de Medicina de Botucatu, Programa de Pós-graduação em doenças infecciosas, Botucatu, SP, Brasil.; 3 Universidade Estadual Paulista, Faculdade de Medicina de Botucatu, Departamento de doenças infecciosas, Botucatu, SP, Brasil.

**Keywords:** COVID-19, Epidemiology, Prevention, Masks, Lockdown

## Abstract

Even though most current recommendations include the general use of masks to prevent community transmission of SARS-Cov-2, the effectiveness of this measure is still debated. The studies on this policy include physical filtering tests with inanimate microparticles, randomized clinical trials, observational studies, ecological analyses, and even computational modeling of epidemics. Much of the so-called evidence is inferred from studies on different respiratory viruses and epidemiological settings. Heterogeneity is a major factor limiting the generalization of inferences. In this article, we reviewed the empirical and rational bases of mask use and how to understand these recommendations compared to other policies of social distancing, restrictions on non-essential services, and lockdown. We conclude that recent studies suggest a synergistic effect of the use of masks and social distancing rather than opposing effects of the two recommendations. Developing social communication approaches that clarify the need to combine different strategies is a challenge for public health authorities.

## INTRODUCTION

The need for urgent measures to control the COVID-19 pandemic is compelling scientific research to rapidly produce results that support public policies^1^. Scientific inferences and expert opinions have been immediately translated into recommendations, which often change in weeks. The use of masks by the general population is a typical example^2^. Given the risk of shortage of personal protective equipment (PPE) for healthcare workers (HCWs) in the early stages of the pandemic, the World Health Organization (WHO) emphasized the “lack of evidence” on the benefit of the use of masks by the general population^3^. This view of the WHO led to severe criticism, and the guidelines changed extensively over the weeks. Still, the organization was especially careful in using the term “advice” rather than “recommendation” for the universal use of cloth masks^4^. 

Despite the success of the popular “#mask4all” campaign (https://masks4all.co/), preventive policies must rely on something stronger than public opinion. Herein, we reviewed the scientific results on the effectiveness of masks for preventing SARS-Cov-2 transmission in community settings; additionally, the current “evidence” is critically appraised and the rationale behind the inclusion of this strategy in public health policies is provided.

## THE FUNDAMENTAL QUESTION: HOW IS SARS-COV-2 TRANSMITTED?

Due to the current absence of a vaccine or effective chemoprophylaxis, prevention of COVID-19 relies on so-called “non-pharmaceutical” measures^5^. These strategies are aimed at breaking epidemiological chains, and are, therefore, highly dependent on the knowledge about the routes of SARS-Cov-2 transmission.

Extensive research has focused on this issue^6^. However, to be able to propose effective ways of prevention, we must first differentiate between the dominant transmission routes responsible for maintaining and expanding the pandemic and other routes that theoretically play secondary (if any) epidemiological roles.

The latter category involves fecal-oral transmission and contamination routes through inanimate fomites. The possibility of acquiring SARS-Cov-2 from surfaces and clothes has been supported by the persistence of viable viruses, documented over a period ranging from hours to several days^7^. However, the presence of pathogens on a surface is only one of several criteria required to ascertain that fomites are epidemiologically relevant, and there is no substantial evidence linking that route of transmission with individual cases or outbreaks^6^ ^,^ ^8^. Given the severe economic implications (such as extensive advertising and selling of sanitizers) and the likelihood of a deleterious shift of focus in preventive measures, the United States Centers for Diseases Control and Prevention (CDC) has stated that “touching a surface or object that has the virus on it and then touching their own mouth, nose, or possibly their eyes (…) is not thought to be the main way the virus spreads (…)”^9^. Having said this and recognizing the paucity of reports on mother-to-child transmission (e.g., vertical transmission through breastfeeding) or acquisition through blood transfusion^6^, we can move on to respiratory droplets and aerosols.

The topic of transmission is of crucial importance in healthcare settings. Current isolation precautions and recommendations are classified differently for transmission from respiratory droplets and aerosols^10^. Droplets usually have diameters greater than 5-10 μm and spread over a distance of approximately 1 m when an infected person speaks, sneezes, or coughs. Therefore, close proximity is required for transmission. Furthermore, the use of surgical masks by healthy persons suffices to ensure their safety (and this is of major importance in healthcare settings). In aerosol (airborne) transmission, very small droplets (<5 μm) from the infected persons spread over large distances and remain suspended in the air for a long time. In healthcare settings, this leads to the need for healthy workers and visitors wearing N95 masks and undergoing preferential isolation in negative-pressure rooms^6^ ^,^ ^10^. 

It is generally accepted that SARS-Cov-2 spreads primarily through droplets and occasionally through aerosols mainly produced after healthcare procedures involving the airways^3^ ^,^ ^4^. It is unclear if aerosols can be generated in the community setting, but some activities such as singing and performing physical exercise have been suspected to promote airborne transmission^6^. 

## FILTERING PROPERTIES: LESSONS FROM PHYSICAL TESTING

Though the use of masks dates back to the archetypical “Plague Doctor”^11^ and was extensively documented during other epidemics^12^ ^,^ ^13^, studies on the physical characteristics and filtering capacity of different fabrics are rather recent. Not surprisingly, research conducted in the past 2 decades has focused on the properties of N95 respirators^14^ ^,^ ^15^ ^,^ ^16^ and surgical masks^17^. The occupational risks posed by droplets (e.g., *Neisseria meningitidis* ^18^) or aerosol-transmitted (e.g., *Mycobacterium tuberculosis* ^19^) pathogens have been matters of concern since the late 20^th^ century. This concern was amplified with the emergence of avian influenza (H5N1) and the H1N1 pandemic in 2009^20^. It is also worth noting that HCWs were highly affected during the SARS outbreaks in 2003^21^. These aspects, together with the obvious requirement of healthy doctors and nurses caring for patients during epidemics and pandemics, justify the focus on the filtering properties of N95 and surgical masks in hospitals and outpatient units^14^ ^,^ ^15^ ^,^ ^16^. An interesting review of research methods and results was published by Rengasami et al^22^. Since HCWs are of secondary importance to our article, we will discuss a single and exemplary study. In light of previous research on inanimate particles^23^, Balazy et al.^17^ examined the efficacy of two types of N95 respirators and two types of surgical masks using aerosols containing the bacteriophage MS2. The aerosol particles had different sizes and were tested in two airflow velocities: 30 L/min (simulating HCW breathing during light workload) and 85 L/min (simulating HCW inhaling during heavy workload). They found a significantly high filtering capacity with N95 respirators. However, even those respirators allowed the penetration of a significant quantity of viruses when expelled in the form of very small particles or when challenged with 85 L/min airflow. Thus, any type of protective mask, no matter the certification, is far from being absolutely safe^4^ ^,^ ^22^ ^,^ ^23^.

It has been widely stated by health agencies that the use of surgical masks of respirators by the general population can lead to shortage of these resources in healthcare settings^3^ ^,^ ^4^ ^,^ ^9^. Besides health agencies, even the popular “#mask4all” campaign (https://masks4all.co/) has recommended the use of cloth masks in community settings. This opens avenues for studies focusing on the filtering capacity of various types of fabrics used in the manufacture of homemade masks.

In the immediate pre-pandemic era (i.e., in 2019), Neupane et al.^24^ performed an optic microscopic analysis of cloth masks and compared the findings with those of surgical masks. To determine the filtering efficiency, they wrapped petri dishs with the masks and exposed them to environmental air (in central Katmandu, Nepal) and counted the particles on each side of the fabric afterwards. The filtering efficiency ranged from 63% to 84% for cloth masks and 94% for surgical masks. They were significantly associated with the density and size of the pores in each fabric. In addition, the repeated performance of washing and drying masks altered the pore size and decreased the filtering efficiency by more than 25%. Thus, the lack of standardized protocols on fabrics and particle size could lead to the possible overestimation of mask efficiency. However, the decline in filtering capacity with the processes of washing and drying must be considered when advising the public to use cloth masks during the COVID-19 pandemic.

Studies conducted during the COVID-19 pandemic have assessed the filtering properties of cloth masks made of different types of fabrics^24^ ^,^ ^25^ ^,^ ^26^. A schematic view of the operational aspects and results is presented in Table 1. Briefly, efficiency varied from 5% to more than 90% and was associated with the fabric and the number of layers in the mask. We must understand those findings in the current view of *emergency science* ^1^ ^,^ ^27^, that is, recommendations can (or must) change as new research data become available. 

**TABLE 1:** Summary of methodological aspects and results of selected studies conducted during COVID-19 pandemic, addressing filtering efficiency of cloth masks

Reference	Fabrics	Methods	Relevant results
Konda et al.^24^	N95, surgical masks cotton, chiffon, flannel natural silk, nibrids (cotton/chiffon, cotton/silk, cotton/flannel)	Mechanical challenge with NaCl aerosols, at 2 cubic feet per minute (CFM) flow rate	Filtering efficacy was generally high for particles greater than 300 nm. Filtering properties of N95 respirators and surgical masks for particles measuring less than 300 nm fell from 85±15 to 34±15 and 76±22 to 50±7, respectively, when gaps were present (a situation similar to innapropriat fitting). For other fabrics, the filtering efficacy increased with the number of layers and was high for cotton/chiffon, cotton/silk and cotton/flannel hybrid masks.
Lustig et al.^25^	N95, Cellulose-filter masks, white denim, cotton (both original fabrics and made from clothes), flannel	Mechanical challenge with virus-like inanimate particle aerosols, 14 L/min flow	Results were compared with the efficacy of N95 respirators. *Higher efficacy* **:** cellulose filter masks, two-layer denim, hybrid fabrics containing four-layer Kona cotton. *Similar efficacy*: four-layer Kona cotton, two-layer fabrics (cloth tower, white flannel, heavy 100% cotton T-shirt, flannel lab coat, and other hybrid fabrics). *Lower efficacy*: three or fewer layers of Kona cotton, one-layer fabrics (cotton, flannel, propylene, or hybrid).
Zhao et al.^26^	Propylene used in healthcare workers’ personal protective equipment. Household materials (cotton from pillow cover, T-shirt and sweater), polyester (from toddler wrap), silk (from napkins), nylon (from exercise cloths), cellulose (from paper towels, tissue paper and copy paper)	Mechanical challenge with NaCl aerosols, at flow rate of 32 L/min. Filtering efficacy measured for different times of exposure	Most common fabric presented low-to-moderate filtering efficacy for short periods. That efficacy can be increased by fabric density (g/m^2^), or decreased with humidity or changes in pore sizes due to washing and drying.

## WHAT CAN WE LEARN FROM ANIMAL EXPERIMENTS?

Animal studies on the transmission of influenza virus have been conducted since the 1930s^28^. Most animal models use ferrets, and studies have supported several routes of transmission: direct contact, indirect contact (fomites), droplets, and aerosols^29^. Similar findings have been reported for other viruses, including coronaviruses^30^ ^,^ ^31^. The controversial issue here is whether exposure in animal studies mimic the real-life situations of human beings. In other words, forced prolonged contact, generating aerosols with air turbulence, and other laboratory strategies are often regarded as being too artificial to have their findings translated into epidemiological policies^29^.

 A recent study conducted by Chan et al.^32^is of particular interest to our discussion. Briefly, the authors placed SARS-Cov-2-infected hamsters and naïve hamsters in adjacent cages. Some cages were separated from each other by a fabric similar to that used in surgical masks. The “surgical mask” partition reduced transmission among hamsters from 66% to 25%. This is an interesting finding, but two important limitations remain: (i) the concerns regarding the “artificiality” of exposure in the model and (ii) the focus on surgical masks, which have not been approved for use by the general population. However, given the ethical limitations in conducting studies on SARS-Cov-2 involving human subjects, “pre-clinical” research on this topic is welcome.

## CLINICAL STUDIES: ADVANTAGES AND LIMITATIONS OF ANALOGY

Studies conducted with humans (i.e., clinical studies) are of utmost importance. Therefore, both observational studies and intervention research on the efficacy of masks have been the subject of systematic reviews (SRs) in the last few months. Acknowledging their valuable efforts, this section relies heavily on the reviews conducted by McIntyre & Chungtai^33^, Liang et al.^34^, Chou et al.,^35^and Chu et al.^36^ Their search strategy, inclusion criteria, methods of analysis, and conclusions are presented in Table 2.

**TABLE 2:** Selected systematic reviews of clinical studies addressing the effectiveness of mask use in the community

Reference	Studies included	Literature bases	Interventions	Outcomes	Meta-analysis	Findings
MacIntyre & Chughtai^33^	8 RCTs on the use of masks by susceptible exposed healthy persons in the community	MedLine Embase	6 RCTs: Surgical masks or PFF2 respirators 2 RCTs: Cloth masks (In 7 RCTs, efficacy of hand hygiene was also tested)	Laboratory-confirmed influenza Influenza-like illnesses	No	Two studies found efficacy of surgical masks and PF2 respirators in subgroup analysis (but not intention-to-tread analysis). Four studies found varied efficacies of masks when combined with hand hygiene intervention, but not for masks alone. Two studies did not find any significant impact of mask use.
	5 RCTs on the use of masks by sick persons		All studies involved use of surgical masks	Laboratory-confirmed influenza Influenza-like illnesses Seasonal coronavirus	No	One study found efficacy of masks. One study found efficacy associated with adherence, but not in intention-to-treat analysis. Three studies found no impact of mask use.
Liang et al.^34^	5 RCTs 3 observational studies All studies listed above involved use by susceptible exposed persons in the community	PubMed, Web of Science, Cochrane Library Chinese National Knowledge Infrastructure (CNKI) VIP (Chinese) database	Even though some studies included other interventions, only the isolated impact of mask use was analysed. 6 out of 8 studies included involved the use of surgical mask.	Laboratory-confirmed respiratory virus infection	Yes	Even though only three studies found protective impact, the meta-analysis found an overall risk ratio [RR] of 0.43 (95%confidence interval [CI], 0.36-0.79). The findings suggest an average 47% protection.
Chout et al.^35^	12 RCTs 3 observational studies	Multiple electronic databases, including the World Health Organization COVID-19 database and medRxiv preprint server	Use of masks (mostly surgical)	RCTs: Laboratory-confirmed influenza, Influenza-like illnesses Observational: SARS-1, MERS, SARS-Cov-2	No (but rigorous analysis of quality of evidence and biases conducted)	1 out of 12 RCTs found some evidence for protection against respiratory viruses. Observational studies supported some protection against SARS-1 and MERS, but evidence for SARS-Cov-2 is still lacking.
Chu et al.^36^	No RCT 3 observational studies	MEDLINE, PubMed, Embase, CINAHL the Cochrane Library, COVID-19 Open Research Dataset Challenge, COVID-19 Research Database (WHO), Epistemonikos EPPI Centre living systematic map of the evidence, ClinicalTrials.gov, WHO International Clinical Trials Registry Platform, (also relevant documents on the websites of governmental and other relevant organisations, reference lists of included papers, and relevant systematic reviews)	Several nonpharmaceutical interventions were analyzed, but only masks were of interest to our review. In all studies relevant to this review (i.e., studies in the community setting), the overall use of masks (regardless of mask type) is analyzed	All comparative studies focused on SARS-1(No study focusing on MERS or COVID-19)	Yes	2 studies found protective effect of mask use. The meta-analysis found overall RR of 0.56 (95%CI, 0.40-0.79). Average protection of 44%.

Note: Most systematic reviews included studies in healthcare settings, but only reviews of studies in the community were included in this table. There are obviously considerable overlaps (i.e., the same studies were included in different systematic reviews). However, the strategies, findings, and conclusions demonstrate that the results are highly dependent on *a priori* assumptions. Notice that MacIntyre & Chughtai^33^ also analyzed studies on mask use by sick persons (i.e., source control).

All SRs presented here were conducted rigorously. However, their rationales, research questions, searching strategies, interventions, and analysis methods (i.e., models of meta-analysis and measures of quality indicators) vary widely. Most importantly, the outcomes of included studies vary widely, including infection by several laboratory-confirmed respiratory viruses (mostly influenza, but also coronaviruses) and clinically defined influenza-like-illnesses (ILIs). Given these limitations, our trust in their conclusions and practical recommendations rely mostly on analogical reasoning.

Rothman argued that the principle of analogy of Bradford Hill’s causation criteria is the weakest, as it is highly (and perhaps inappropriately) influenced by the researchers’ creativity and imagination^37^. This heterogeneity also exemplifies what Stegenga^38^ termed "the malleability" of clinical research, which limits its claim of objectivity and the strength of its evidence in SRs. However, if analyzed with a rational filter, the studies presented above provide interesting insights into the use of masks. Of note, some studies identified the protective effects of early use and good adherence to the use of masks^33^ ^,^ ^34^ ^,^ ^35^.

## ASKING PROPER QUESTIONS TO GET PROPER ANSWERS

Most studies included in the SR cited in the previous section^33^ ^,^ ^34^ ^,^ ^35^ ^,^ ^36^carefully assessed the confounding factors, such as baseline influenza vaccination and intensity of exposure to infected persons. However, individual-based studies (except cluster-randomized trials) did not provide good adjustments for population-level exposures and outcomes. In this setting, results from ecological studies^39^ and modeling^40^ may be of special interest.

Zhang et al.^41^ addressed the routes of SARS-Cov-2 spread and the effectiveness of masks by comparatively analyzing the time trends of COVID-19 in New York state and the USA. Briefly, the authors applied interrupted time series (ITS) analysis to the trends before and after three population-level interventions: (i) social distancing (recommended for the whole country in March 2020), (ii) stay-at-home recommendation (applied in March in New York and in April in the whole of the USA), and (iii) face-covering (generally by masks) recommendation (applied in New York only on April 17^th^, 2020). The results showed the impact of “stay-at-home”, which was further increased by the face mask recommendation. Further comparisons with time trends in Wuhan (China) and Italy supported the primary impact of mask use, in conjunction with simultaneous social distancing, quarantine, and contact tracing.

Another interesting methodological approach was devised by Howard et al.^42^, i.e., mixing SR of ecological data with computational modeling of the impact of masks on the basic reproduction number (R_0_) of the SARS-Cov-2. Briefly, the results indicate a synergistic effect of the physical filtering properties of mask fabrics and the proportion of the population who showed adherence to its use. In an optimal scenario, this would represent a decrease in initial R_0_ from 2.4 to a post-intervention reproductive number (R_e_) below 1. As one may be aware, sustained R_e_ below 1 will lead to a decrease in cases and, ultimately, end the outbreak^42^. Still, Howard et al. recommend this measure in conjunction with other nonpharmaceutical strategies.

Both Zhang’s^41^ and Howard’s^42^ approaches do not fit well in the Cochrane methodological hierarchy of generating evidence^27^ ^,^ ^43^. However, by addressing population-level interventions and outcomes, they provide an interesting rationale for public health strategies. Most importantly, both authors recommend the use of masks “in conjunction with widespread testing, contact tracing, quarantining of anyone that may be infected, hand washing, and physical distancing…face masks are a valuable tool to reduce community transmission.”

This type of methodological humility is particularly appropriate; it emphasizes the importance of asking the right question. In brief, despite strong statements made by Zhang et al.^41^, no study posed a research question like “can mask use be recommended instead of social distancing/lockdown policies?” This question is hard to address even considering the extensive data that may support natural experiments. A reasonable question (e.g., “is mask use a good public health strategy in conjunction with distancing policies?”) is likely to produce answers that guide policies directed at communities, while preventing a false sense of safety (with mask use) that exposes people to high levels of risk (e.g., crowding in close places).

## RATIONALE AND PRACTICAL ADVICE

Based on the discussion presented above, one may infer that the WHO is concerned about the false sense of safety and possible shortage of personal protective equipment for HCWs^3^ ^,^ ^4^. Still, there is a rational argument for the universal use of masks in the community. Cloth masks may have a reasonable (though far from complete) protective impact that depends on the fabric and number of layers and is probably reduced with successive washing and drying. Based on these findings, reasonable advice for the general population is provided in Table 3. The advice complies with (but is not a copy of) WHO guidelines^3^ ^,^ ^4^. It should be understood as a provisional guide for public policies, which can be modified in the face of novel scientific findings. Novel findings and technologies are particularly welcome. An interesting example is that of “elastomeric masks” that are made of synthetic or natural rubber and can be washed and reused^44^. Even though agencies such as the United States Food and Drug Administration (FDA) still raise concerns about its use, they have been approved for healthcare settings as substitutes for N95 respirators during mask shortages^45^. Since the current review focused on the use of masks outside healthcare settings, those interested in the research on elastomeric respirators can refer to recent articles and guidelines^44^ ^,^ ^46^.

**TABLE 3:** General advices for manufacture and use of masks in community settings^3^.

Prevention aspects	Advices
Physical properties	● Masks should have at least two layers of fabric (preferably cotton, tricoline or nylon).
	● They should have elastics to attach or straps to tie, ensuring a secure and firm fit close to the face.
	● Reasonable approximate measurements of the fabric are 21 cm high by 34 cm wide. However, they can vary according to the size of the face, and must cover the nose and mouth.
Mask handling	● Before putting the mask on, one should perform hand hygiene (alcohol-gel hub or extensively washing with water and soap).
	● Masks and for individual use and must not be shared.
	● While wearing the mask, one should avoid touching it.
	● The mask should be used for a maximum of two hours. After that time, it should ideally be changed (It is advised that for long exposures at least two masks are available).
	● In case of exchange, the used mask should be kept in a plastic waterproof bag.
	● The mask should be removed from the back to the front, never touching the outer surface.
	● Used masks should be washed with soap and water and ideally soaked for 20 minutes in hypochlorite (bleach) of 2 to 2.5% concentration. It is advised that masks are discarded after being washed several times.
	● Masks should be discarded whenever it shows signs of deterioration or impaired functionality.
Complementary issues	● Maintain social isolation and respiratory etiquette, covering the mouth and nose with the inside of the elbow when coughing or sneezing.
	● Kisses, hugs and handshakes should be avoided.
	● People with flu-like conditions must stay home (except for medical care, in which case they must wear surgical masks).
	● Under no circumstances should the cloth mask be used by health professionals in dealing with suspected or confirmed cases of COVID-19.

## FINAL REMARKS

The title of this review is a pastiche of one of Raymond Carver’s (1938-1988) famous works, “What we talk about when we talk about love”^46^. In that text, Caver encourages the reader to reflect on an excessively trivialized theme through the demonstration of (somewhat bizarre) situations in which love is expressed. Likewise, this review did not aim to provide exhaustive data on the topic and did not follow the guidelines for SRs or scoping reviews. Instead, we attempted to outline a multifaceted approach to scientific research that supports the use of masks by the general population. Since protection of HCWs was not the focus of this review, some discussions (e.g., reprocessing surgical masks or N95 respirators^47^) were not included in our discussion.

Our provisional conclusion is that there are more reasons for than against the use of cloth masks. The empirical findings are heterogeneous (and highly dependent on the fabrics used to manufacture masks), but the rational support for this strategy is stronger than, for instance, that for recommendations on disinfection of inanimate surfaces^6^ ^,^ ^7^ ^,^ ^8^. However, for the sake of intellectual honesty, we choose the term “advice” over “recommendation,” similar to the WHO. Novel findings on the extraordinary natural experiment that the pandemic has introduced will surely increase our knowledge on this subject.
